# Abscisic Acid: A Conserved Hormone in Plants and Humans and a Promising Aid to Combat Prediabetes and the Metabolic Syndrome

**DOI:** 10.3390/nu12061724

**Published:** 2020-06-09

**Authors:** Mirko Magnone, Laura Sturla, Lucrezia Guida, Sonia Spinelli, Giulia Begani, Santina Bruzzone, Chiara Fresia, Elena Zocchi

**Affiliations:** 1 Nutravis S.r.l., Via Corsica 2/19, 16128 Genova, Italy; 2 Department of Experimental Medicine, University of Genova, 16132 Genova, Italy; laurasturla@unige.it (L.S.); l.guida@unige.it (L.G.); sonia.spinelli94@libero.it (S.S.); giulia.begani@gmail.com (G.B.); santina.bruzzone@unige.it (S.B.); 3 Molecular and Cell Biology Laboratory, Salk Institute for Biological Studies, 10010 N Torrey Pines Rd, La Jolla, CA 92037, USA; chiara.fresia.8@gmail.com

**Keywords:** abscisic acid, prediabetes, type 2 diabetes mellitus, metabolic syndrome, insulin resistance, adipocyte browning, AMP-activated protein kinase, food supplement

## Abstract

Abscisic acid (ABA) is a hormone with a very long evolutionary history, dating back to the earliest living organisms, of which modern (ABA-producing) cyanobacteria are likely the descendants, well before separation of the plant and animal kingdoms, with a conserved role as a signal regulating cell responses to environmental challenges. In mammals, nanomolar ABA controls the metabolic response to glucose availability by stimulating glucose uptake in skeletal muscle and adipose tissue with an insulin-independent mechanism and increasing energy expenditure in the brown and white adipose tissues. Activation by ABA of AMP-dependent kinase (AMPK), in contrast to the insulin-induced activation of AMPK-inhibiting Akt, is responsible for stimulation of GLUT4-mediated muscle glucose uptake, and for the browning effect on white adipocytes. Intake of micrograms per Kg body weight of ABA improves glucose tolerance in both normal and in borderline subjects and chronic intake of such a dose of ABA improves blood glucose, lipids and morphometric parameters (waist circumference and body mass index) in borderline subjects for prediabetes and the metabolic syndrome. This review summarizes the most recent results obtained in vivo with microgram amounts of ABA, the role of the receptor LANCL2 in the hormone’s action and the significance of the endowment by mammals of two different hormones controlling the metabolic response to glucose availability. Finally, open issues in need of further investigation and perspectives for the clinical use of nutraceutical ABA are discussed.

## 1. Introduction

### 1.1. Abscisic Acid (ABA), A Stress Hormone in Plants and Animals

**Figure 1:**
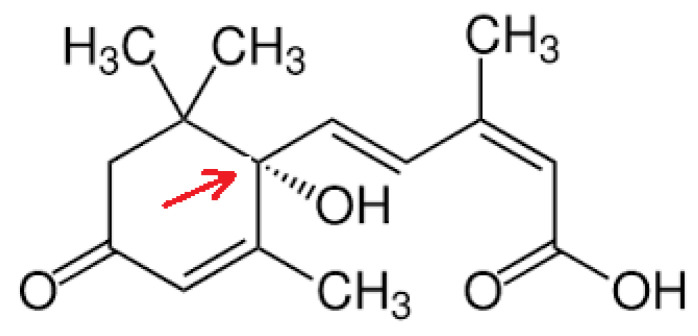
Structure of ABA. 2-cis, 4-trans-Abscisic acid has an asymmetric carbon atom (arrow) generating two enantiomers S-(+)-ABA and R-(-)-ABA. (+)-ABA is the naturally-occurring form in plants, although (-)-ABA is active in some vegetal functional assays [2].

2-cis, 4-trans-Abscisic acid (ABA) is a 15-carbon weak acid (pKa 4.8) terpenoid hormone (Figure 1) that regulates several pivotal physiological functions in plants, mainly involved in the response to abiotic and biotic stress (water and nutrient availability, UV irradiation; pathogen attack) [1].

The function of ABA as a stress signal and its signaling pathway are conserved in all plants, including mosses, and are believed to be the result of the very early adaptation of life to the terrestrial environment. Although the study of ABA in plants has been ongoing for several decades, also in view of its industrial application to improve stress tolerance in crops, it is only in the past decade that evidence has mounted regarding the presence and physiological significance of ABA also in animals. Le Page-Degivry et al. first described the presence of ABA in mammalian tissues, particularly brain [3], but their interesting observation remained isolated. Our group became interested in ABA in our quest for the animal hormonal signal upstream of the second messenger cyclic ADP-ribose (cADPR), a universal Ca^2+^ mobilizer from intracellular stores that had been discovered to be involved in insulin release [4]. Intracellular Ca^2+^ movements are arguably the most ancient and conserved signaling mechanism throughout the animal and plant kingdoms. Indeed, cADPR had been reported to mediate the effect of ABA in guard cells [5]. Thus, we hypothesized a role for ABA as an animal, as well as a plant hormone, and started our investigation on the simplest and most ancient Metazoa, marine sponges and hydroids; the physiological functions of these animals are limited to water filtration and respiration. However, being sessile, they are particularly exposed to environmental stress (e.g., changes in water temperature or light exposure). We discovered that temperature and light stimulated ABA production in sponges and hydroids, respectively, and that ABA stimulated specific functional responses to these environmental challenges (an increase in water filtration and oxygen consumption in sponges, tissue regeneration in hydroids). These functional responses were mediated by a signaling pathway sequentially involving cAMP, PKA, cADPR and intracellular Ca^2+^ movements [6,7,8]. These discoveries warranted further investigations into the role of ABA in cell-specific functions of higher Metazoa.

### 1.2. Micromolar ABA Has Pro-Inflammatory and Insulin-Releasing Activity In Vitro and In Vivo

In several different innate immune cell types exogenous ABA elicits pro-inflammatory functional responses and endogenous ABA is released when cells are challenged with environmental (physical, chemical) stimuli. Briefly, human granulocytes release ABA in response to heat stress or phorbol myristate acetate and exogenous micromolar ABA stimulates cell migration, phagocytosis, ROS and NO production [9]. ABA is released by rat alveolar macrophages stimulated with quartz particles and exogenous micromolar ABA induces the release of pro-inflammatory PGE2 and TNF-α [10]. Micromolar ABA stimulates NO and TNF-α production and cell migration of the mouse microglial cell line N9 and endogenous ABA is released when cells are challenged with bacterial lipopolysaccharide, phorbol myristate acetate, the chemoattractant peptide f-MLP, or β-amyloid [11]. Thus, a plethora of data suggest that ABA in the micromolar concentration range has pro-inflammatory effects on mammalian innate immune cells: a conserved signaling pathway involves the activation of the ADP-ribosyl cyclase CD38 and the production of the Ca^2+^-mobilizing second messenger cADPR. Starting from the observation that cADPR was involved in the Ca^2+^ signaling leading to insulin release, the effect of ABA on β-pancreatic cells was explored. Indeed, micromolar ABA stimulated insulin release in vitro, from rat insulinoma cells and from isolated human islets [12], and also in vivo, in the perfused rat pancreas [13]. In the in vitro study, the role of cADPR as the second messenger of ABA was confirmed, similarly to what observed on innate immune cells.

### 1.3. ABA Is an Endogenous Mammalian Hormone: Nanomolar ABA Peaks in Human Plasma after Glucose Load

The studies described above provided ample demonstration that ABA exerts cell-specific functional effects on mammalian cells through the Ca^2+^-mobilizing second messenger cADPR. However, a clear indication that ABA is indeed an endogenous animal hormone was still missing. In their study, Le Page-Degivry et al. observed that ABA was present in the brain of mice fed a synthetic, ABA-free diet at even higher concentrations compared with mice fed a normal (ABA-containing) chow, arguing in favor of the endogenous origin of ABA [3]. A credible indication that ABA is indeed an endogenous animal hormone came from the observation that plasma ABA increases after a glucose load in normal human subjects, reaching concentrations in the low nanomolar range [14]. Moreover, in the same study, the authors showed that nanomolar ABA stimulated glucose uptake, quantitatively similarly to insulin, in rat L6 myoblasts and in murine 3T3-L1 cells differentiated to adipocytes, by increasing GLUT-4 translocation to the plasma membrane. This study provided the first evidence that endogenous ABA could be involved in glycemia homeostasis at hormone-like concentrations.

### 1.4. ABA Reduces Glycemia without Increasing Insulinemia

The observations that nanomolar ABA stimulates glucose transport in myocytes and adipocytes in vitro and that endogenous nanomolar ABA increases in the blood of normal subjects after glucose load suggested a role for endogenous ABA in the control of glycemia. Insulin and GLP-1 are the only two peptide hormones known so far to stimulate tissue glucose uptake under conditions of hyperglycemia, and GLP-1 acts by stimulating insulin release. Thus, insulin represents a sort of bottleneck in the response to high blood glucose, apparently a weak spot in glycemia control and a conspicuous example contrary to the principle of redundancy that governs most (if not all) biochemical mechanisms fundamental for cell and organism survival. These considerations suggested to explore the possibility that the effect of ABA on tissue glucose uptake could occur independently of insulin. Several experiments were performed in vivo and ex vivo to address this issue.

In a study performed on rats, the animals were administered an oral glucose load without (control) or with synthetic ABA (at a dose of 1 µg/Kg body weight), or with an aqueous fruit extract yielding the same dose of ABA. The animals receiving the extract and those treated with synthetic ABA showed similar and significantly lower values of the area under the curve (AUC) of the glycemia and insulinemia profiles compared with the control group, suggesting that the active molecule present in the extract and responsible for the observed metabolic effects was indeed ABA [15]. The same fruit extract, providing a dose of approximately 0.5 µg/Kg body weight, was also tested on human volunteers as an adjunct to carbohydrate-rich meals (breakfast and lunch): a significant reduction of the AUC of glycemia and of insulinemia was observed in each subject when the meals were taken with the extract compared with the same meals taken without extract [15]. The plasma ABA concentration measured after intake of the extract in humans increased significantly compared with basal (fasting) values for several hours, but remained in the nanomolar range. The sparing effect on insulinemia of ABA at a dose of 0.5–1 µg/Kg was unexpected, since micromolar ABA had been reported to stimulate insulin release from human β-pancreatic cells and from rat insulinoma cells in vitro [12]. Indeed, when rats were subjected to an OGTT with synthetic ABA at 100 mg/Kg, the AUC of insulinemia was not reduced compared with controls, indicating that at this pharmacological dose ABA did not spare insulin release, although it reduced glycemia [15], in line with previously reported results obtained on mice [16].

The most convincing evidence of the insulin-independent activity of ABA on glycemia control was derived in ex vivo experiments: in mouse skeletal muscle samples taken from fasted animals uptake of ^18^F-deoxyglucose (FDG) increased two-fold in the presence of nanomolar ABA compared with untreated controls [17]. Stimulation by nanomolar ABA of muscle glucose uptake was confirmed in vivo, on rats undergoing an oral glucose load; as detected by micro-PET, animals receiving a dose of 1 µg/Kg ABA with the glucose load showed a two-fold increase of FDG uptake in skeletal muscle and consequently a significantly reduced glycemia profile after glucose load [17].

A reduction of plasma insulin levels in response to a carbohydrate load would be desirable since insulin stimulates the conversion of glucose into triglycerides in the adipose tissue. Indeed, hyperinsulinemia, either endogenously produced as a result of reduced insulin sensitivity, or exogenously administered as an anti-diabetic drug, is a major factor contributing to an increase in body weight and hepatic steatosis in the pre-diabetic and diabetic patient [18]. Could (endogenous) ABA be a safeguard against excess insulin release?

An indication that ABA can substitute in part for insulin comes from studies on insulin-deficient mice. Administration of 1 µg/Kg ABA together with an oral glucose load reduced the AUC of glycemia in wild-type and also in TRPM2^−/−^ mice [17], which have a significantly reduced insulin secretion in response to glucose [19]. The AUC of insulin during the OGTT with ABA was also significantly reduced in the TRPM2^−/−^ animals, as in the wild-type mice, compared with the respective OGTT without ABA [17], indicating that the effect of ABA was independent of insulin in both genotypes. Finally, the daily intake of 1 µg/Kg ABA increased muscle glycogen content in TRPM2^−/−^ mice fed a high-glucose diet similarly to what observed in wild-type animals, indicating that ABA stimulated muscle glucose uptake also in insulin-deficient animals.

### 1.5. ABA Improves Lipidemia and Reduces Body Weight and Cardiovascular Risk in Borderline Subjects

In a recently published human study, the daily intake of a food supplement containing a vegetal extract titrated in ABA in its composition GSECM-50^®^, sufficient to yield a dose of ABA of approximately 1 µg/Kg body weight, for 75 days significantly improved the metabolic (fasting glycemia, glycated hemoglobin, total, LDL and HDL cholesterol) and morphometric parameters (waist circumference and body mass index) currently employed to evaluate the risk for metabolic syndrome and diabetes. At the end of treatment, fasting blood glucose, glycated hemoglobin, total, LDL and HDL cholesterol, body mass index and waist circumference had significantly improved compared to values at time zero, particularly in those subjects with starting borderline values. The Framingham point score and the 10-year percentage risk calculated for each subject before and at the end of treatment were both significantly reduced in all subjects. In the same study, the daily intake of the same dose of synthetic ABA for four months improved glucose tolerance, and reduced glycated hemoglobin, blood lipids and body weight in mice fed a high-glucose diet [20]. The human and murine parts of the study, taken together, allow to conclude that the improvement of metabolic and bodily parameters observed in the clinical study, performed with the food supplement, can be attributed to ABA present in the vegetal extract of the composition, because similar results were observed on mice fed the synthetic, pure molecule. In addition, mice have certainly not changed their feeding behavior during the study, allowing to rule out this possibility as an explanation for the improvement of the parameters investigated in both studies.

In another set of experiments, the effect of a single dose of the same ABA-containing food supplement used in the chronic study was tested on the glycemia profile after intake of a standardized carbohydrate-rich breakfast. A significant reduction of the mean glycemia profile and of the mean AUC of glycemia (measured over 120 min) was observed in each subject when breakfast was taken with the food supplement compared with the same breakfast taken without supplement [20]. Intake of the ABA-rich food supplement significantly increased ABAp 5- to 16-fold over fasting levels (5–15 nM), indicating that oral ABA was readily absorbed and contributed to increase endogenous ABAp [20].

**Figure 2:**
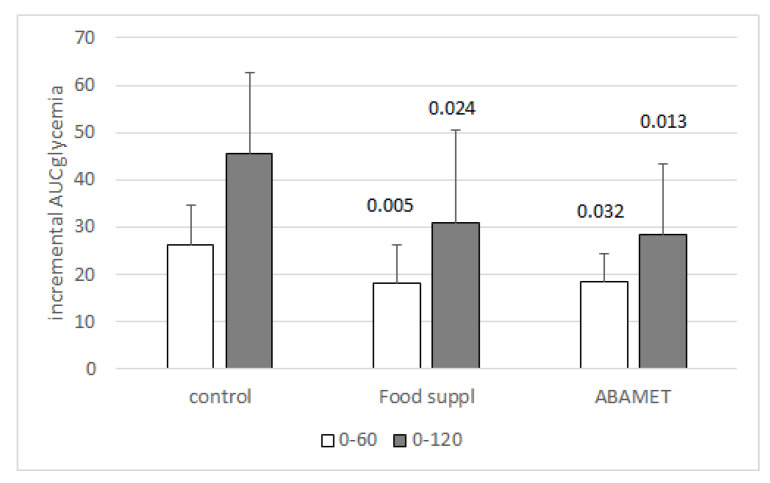
The active ingredient of the food supplement is the ABA-containing vegetal extract. Six volunteers introduced a standardized carbohydrate-rich breakfast, one without any supplement (control), one with one tablet of the formulated food supplement (Food suppl) and another taking only the amount of vegetal extract (ABAMET^®^) present in one tablet of the food supplement. The three experiments were performed one week apart. The food supplement or ABAMET^®^ were taken before the meal [20] Mean ± SD values of the incremental AUC of glycemia in the time frames 0–60 and 0–120 min are shown. *p* values by paired, two-tailed *t*-test of the comparison between each bar with the respective control bar are shown.

A similar significant reduction of the AUC of glycemia as observed with the formulated food supplement was observed when the same standardized carbohydrate-rich breakfast was taken with the ABA-containing vegetal extract alone (ABAMET^®^), indicating that the active ingredient of the food supplement was indeed the ABA-containing vegetal extract (Figure 2).

A daily dose of 0.5–1 µg ABA/Kg body weight, sufficient to improve glucose tolerance in humans in the nanomolar range, is approximately 1 log higher than the amount of hormone that can be ingested daily with a fruit- and vegetable-rich diet [15]. The nutraceutical composition GSECM-50^®^ containing the ABA-rich vegetal extract ABAMET^®^ can provide the optimal daily dose of ABA sufficient to increase endogenous ABAp levels to the extent producing a beneficial effect on human metabolism [20].

### 1.6. ABA Stimulates White Adipocyte Browning and BAT Activity

The reduction in body weight in the face of unchanged dietary habits observed in humans and in mice treated with low-dose ABA could be attributed to the sparing effect of ABA on insulin release; however, adipose tissue also seems to be a direct target of ABA, along with skeletal muscle, as suggested by in vitro and in vivo studies. Unlike insulin, ABA does not induce preadipocyte differentiation into triglyceride-rich adipocytes in vitro; instead, treatment with ABA induces adipocyte remodeling in differentiated cells, reducing cell size and increasing mitochondrial content, O_2_ consumption and expression of the brown adipocyte-specific genes UCP-1, PGC-1α, TMEM26, PRDM16 and CIDE-A. In vivo, a single oral dose of ABA 1 µg/Kg increased BAT glucose uptake 2-fold in rats, as detected by micro-PET, and treatment of mice for 30 days with the same dose significantly increased expression of BAT genes in the WAT and in WAT-derived preadipocytes, isolated from the treated animals [21]. Mitochondrial DNA increased 20-fold in the WAT from ABA-treated mice compared with untreated controls and expression of UCP-1 in the BAT was also significantly higher in ABA-treated as compared with control animals [21]. Thus, oral low-dose ABA stimulates BAT activity and induces browning features in the WAT of chronically treated mice. These actions of ABA on AT could be responsible for the observed reduction of body weight in chronically ABA-treated mice compared with controls: female C57Bl/6 mice fed a high-fat diet and treated with ABA (1 µg/Kg body weight/day) for 12 weeks showed a significantly lower weight gain compared with untreated controls, in the face of a higher food intake in the ABA-treated animals: the body weight relative to time zero was 1.30 ± 0.1 vs. 1.53 ± 0.1 in ABA-treated vs. untreated animals (*n* = 5; *p* < 0.01 by two-tailed, unpaired *t* test); the daily food intake during the period of observation was 2.7 ± 0.2 vs. 2.4 ± 0.2 g/animal/day in ABA-treated vs. untreated animals (*n* = 5; *p* < 0.01 by two-tailed, unpaired *t* test) (Magnone M. unpublished result).

### 1.7. The Plasma ABA Response to A Glucose Load Is Impaired in T2D and in GDM

Plasma ABA increases in normal human subjects after an oral glucose load [14], but not in patients with type 2 diabetes mellitus (T2D) nor in pregnant women with gestational diabetes mellitus (GDM). Resolution of GDM one month after childbirth coincides with a restoration of the normal ABAp response to glucose load [22].

Interestingly, a significant increase of ABAp relative to pre-surgical values was observed in obese patients one month after bilio-pancreatic diversion (BPD), a type of bariatric surgery performed to reduce body mass and improve glucose tolerance [22]. The increase of fasting ABAp was observed both in normal glucose tolerant (NGT) and in T2D obese subjects, in parallel with a reduction of fasting blood glucose and a significant decrease of the HOMA-IR and fasting insulinemia in the diabetic subjects [22]. Another difference observed between T2D and NGT subjects regarded the fasting ABAp levels, which were significantly higher in T2D compared with NGT subjects; the respective median values were 1.15 (0.19–4.77) vs. 0.66 (0.13–1.72) (*n* = 21 T2D and 27 NGT; *p* = 0.013 by Mann–Whitney test). Moreover, the distribution of the ABAp values was normal in NGT, but not in T2D subjects [22]. These abnormalities may be caused by a heterogeneity of ABA-related dysfunctions occurring in T2D, such as resistance to the effect of ABA (inducing an increase of ABAp, as occurs with insulin in insulin-resistant subjects), or the inability of ABAp to increase in response to hyperglycemia (causing ABAp levels to be in the normal range despite hyperglycemia).

Collectively, these observations on diabetic patients suggest a role for ABAp dysfunction in the development of glucose intolerance and obesity and a beneficial effect of elevated ABAp on glycemic control; indeed, one can expect that insufficiency of either one of the hormones regulating tissue glucose uptake and its metabolic disposal (insulin and ABA) should negatively affect glycemia control.

### 1.8. The ABA Signaling Pathway Is Different from That of Insulin

Conservation of ABA between plant and animal kingdoms suggested to explore whether the ABA receptor could be also conserved. Among the several receptors identified in different plant tissues, a putative G-protein coupled receptor (GCR2) [23] indeed showed a significant amino acid sequence identity with a mammalian family of proteins, the lanthionine synthetase C-like protein (LANCL) family. GCR2 has since been disputed as a G protein-coupled receptor, and its homology with the bacterial lanthionine synthetase protein superfamily has instead been advocated [24], a homology that also pertains to the mammalian LANCL proteins. Although the current general consensus is that the PYR/PYL/RCAR family of intracellular receptors are the principal ABA receptors in land plants [25], the homology between mammalian LANCL proteins and plant GCR2 suggested to explore the possibility that they were implicated in ABA sensing. A role for the LANCL protein family in lanthionine biosynthesis has since been ruled out [26]. The LANCL family comprises three proteins: LANCL1 is the most highly expressed in mammals, particularly in the brain, and is a cytosolic protein, LANCL2 is membrane-anchored through its myristoylated N-terminal [27] and LANCL3 has the lowest expression levels of the LANCL proteins and appears to be cytosolic. The membrane-bound location of LANCL2 first attracted interest in this protein as a putative mammalian ABA receptor; indeed, several in vitro studies indicate that human recombinant LANCL2 binds ABA with a high affinity (Kd 2.6 nM) [28,29] and is required for ABA action in several different mammalian cell types [30]. LANCL2 has an unusual behavior as a hormone receptor as it is coupled to a G protein when membrane bound, but can also detach from the membrane when de-myristoylated and accumulate in the cell nucleus [27]. Indeed, the nuclear translocation of LANCL2 occurs following ABA binding [31]. This behavior appears to combine features typical of the receptors for peptide (G protein coupling) and for steroid hormones (nuclear translocation), perhaps a heritage of the primordial origin of the hormone, or the result of the solubility features of ABA.

ABA is a weak acid (pKa = 4.8). Protonated ABA can diffuse through the lipid bilayer [32]; however, a very low percentage of ABA is protonated at the near-neutral pH of plasma and interstitial liquid. For this reason, the presence of a transport system is essential for ABA trafficking between extra- and intracellular fluids. Conversely, the strongly acidic pH present in the stomach probably allows the diffusion of protonated ABA through the gastric lipid bilayer, accounting for the rapid absorption of the hormone after oral intake [15].

Binding of ABA to LANCL2 bound to the inner plasma membrane layer requires influx of the hormone through the plasma membrane, which occurs through transporters of the anion exchanger (AE) superfamily [33].

The signaling pathway downstream of LANCL2 has been studied in the target cells of the immune (monocytes, macrophages and T lymphocytes) [34], and of the metabolic actions of the hormone (adipocytes and muscle cells) [17,21]. In innate immune cells, ABA binding to its receptor leads to the activation of adenylate cyclase and the subsequent phosphorylation and activation of the ADP-ribosyl cyclase CD38 by protein kinase A (PKA). The product of the enzyme action of CD38 on its substrate NAD^+^, cADPR, then triggers an intracellular Ca^2+^ rise due to both intracellular Ca^2+^ release from ryanodine-sensitive endoplasmic Ca^2+^ channels and to extracellular Ca^2+^ influx due to ADPR-gated plasma membrane Ca^2+^ channels [9]. This sequence of events closely parallels the ABA signaling pathway first described in marine sponges [6]. The transcriptional effects of ABA observed on hemopoietic progenitors are also likely mediated by the observed increase of intracellular cAMP and the consequent activation of the cAMP-responsive transcription factor CREB [35]. A role for NF*k*B in the micromolar ABA-induced activation of the transcription of cyclooxigenase-2 has also been observed in quartz-stimulated rat alveolar macrophages [10], again pointing to intracellular Ca^2+^ movements as an important feature of the ABA signaling pathway in inflammatory cells. The effect of ABA on cells of the hemopoietic lineage (progenitors and innate immune cells) occurs at low micromolar concentrations, thus it is possible that a different signaling pathway is activated by the low nanomolar concentrations exerting its metabolic actions. The signaling pathway downstream of LANCL2 in macrophages and Treg was studied in silico by Leber et al., suggesting a role for LANCL2 in the induction of regulatory responses in macrophages and T cells during *H. pylori* infection [36].

In adipocytes, stimulation by ABA of glucose uptake via GLUT4 involves the activation of phosphoinositide 3-kinases (PI3K). Interestingly, the N-terminal sequence of LANCL2 has been shown to bind to phosphatidylinositol phosphates (PIPs), particularly PI3P, on the plasma membrane, suggesting a spatial as well as functional correlation between LANCL2-dependent and PI3P-mediated signaling. In muscle cells, AMP-activated protein kinase (AMPK) appears to mediate the nanomolar ABA-induced increase of glucose transport, since the effect of ABA is abrogated by pre-treatment of cultured myocytes and of murine muscle biopsies with the AMPK inhibitor dorsomorphin [17]. Activation of AMPK in the ABA-signaling pathway is in sharp contrast with the signal transduction activated by insulin, which results in the inactivation of AMPK by Akt/PKB-mediated phosphorylation.

Indeed, the effect of ABA on energy metabolism appears to be different from that of insulin, pointing to non-overlapping physiological functions of these hormones.

**Figure 3:**
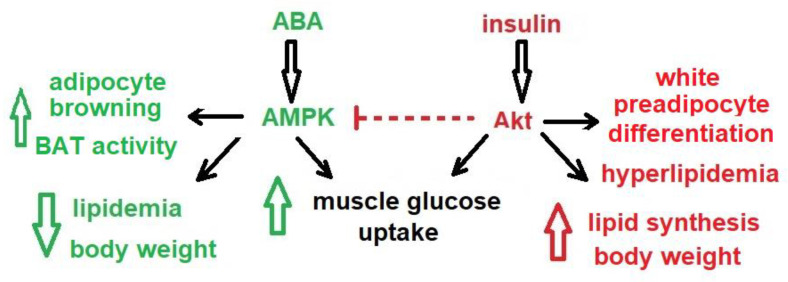
Non-overlapping roles of ABA and insulin in the regulation of energy metabolism. Insulin, via the kinase Akt, stimulates glucose uptake in skeletal muscle and white adipose tissue (WAT), triglyceride synthesis in adipocytes, preadipocyte differentiation into WAT and increased hepatic triglyceride synthesis and lipoprotein export into the blood. Nanomolar ABA, via the AMP-dependent protein kinase (AMPK), stimulates muscle glucose uptake similarly to insulin, but the effect on adipose tissue is different. ABA does not induce preadipocytes differentiation; instead, it stimulates the expression of browning genes in the WAT and increases glucose uptake and mitochondrial uncoupling in brown adipose tissue (BAT). The effect of insulin and of ABA on body weight (BW) is opposite, with insulin inducing an increase and ABA instead favoring a decrease. The relative plasma concentrations of these hormones is likely to affect BW homeostasis and energy metabolism. Via Akt, insulin inhibits AMPK and the metabolic responses to low cell energy levels.

Glucose intake induces an increase of ABAp and one could hypothesize that this hormone provides the first response to nutrient availability, stimulating muscle glucose uptake and thermogenic energy expenditure. Persistence of hyperglycemia, despite the action of ABA and in excess of muscle energy requirement, then results in insulin release and in the activation of the metabolic responses to nutrient abundance (glycogen and fatty acid synthesis, adipocyte differentiation and accumulation of triglycerides) (Figure 3). Interestingly, in 3T3-L1-derived murine adipocytes the siRNA-mediated downregulation of LANCL2 expression reduces both the ABA- and insulin-induced glucose uptake and downregulates Akt phosphorylation after insulin treatment [21], suggesting that levels of LANCL2 expression in adipocytes could affect insulin sensitivity.

Activation by ABA of the transcription and phosphorylation of AMPK opens new perspectives on the signaling pathways activated by the hormone. AMPK phosphorylates and inhibits the transcriptional activity of PPAR-γ, the master regulator of adipogenesis, thereby preventing the differentiation of preadipocytes [37] and triglyceride accumulation [38]. AMPK is also an upstream positive regulator of p38 MAPK [39], which promotes PPAR-γ phosphorylation on Ser122, thus preventing PPAR-γ mediated inhibition of GLUT4 expression [40]. The partial suppression of the transcriptional activity of PPAR-γ in heterozygous PPAR-γ-deficient mice results in an improved insulin sensitivity and in a reduced tendency to obesity [41,42] and mice chimeric for wild-type and PPAR-γ null cells exhibit little or no contribution to adipose tissue formation by null cells [43]. The observations that low-dose ABA significantly reduces body weight in mice fed a high-glucose diet and in humans [20] and improves muscle glucose uptake are in agreement with the activation of AMPK in adipocytes and muscle cells.

### 1.9. LANCL2 Is Not the Only Mammalian ABA Receptor

The role of LANCL2 in mediating the stimulatory effect of ABA on innate immune cell function and on energy metabolism appears to be somewhat different.

**Figure 4:**
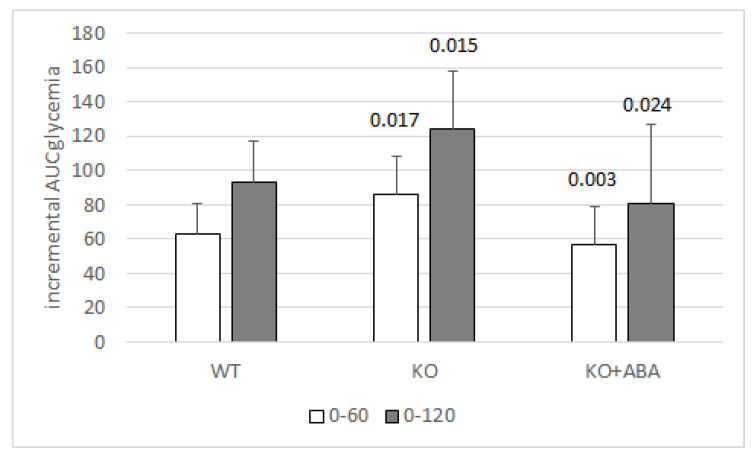
LANCL2^−/−^ mice have a reduced glucose tolerance, but respond to ABA. Eight-hour fasted, male LANCL2^−/−^ (KO) and wild-type (WT) mice (6/group) were subjected to OGTT (2 g glucose/Kg body weight) without or with ABA (1 µg/Kg body weight). Glycemia was measured before gavage (time zero) and at 30, 60 and 120 min thereafter. The incremental AUC of glycemia was calculated in the time frames 0–60 and 0–120 min by the trapezoidal rule on values relative to time zero. *p* values by two-tailed unpaired *t*-test (KO vs. WT) or by two-tailed paired *t*-test (KO + ABA vs. KO). The genetic ablation of LANCL2 in KO mice was confirmed by genotyping. Absence of LANCL2 protein expression in skeletal muscle, liver, WAT and BAT of KO mice was confirmed by Western blot (not shown), (Magnone M., unpublished results).

Unlike inflammatory cells, where LANCL2 silencing abrogates the response to ABA [30] in adipocytes and muscle cells, silencing of LANCL2 reduces, but does not eliminate, the effect of ABA [14,17], suggesting a role for other receptors in the metabolic action of the hormone. A more direct indication that other receptors could contribute to mediate the metabolic actions of ABA comes from studies on LANCL2 knock-out mice. Indeed, in C57Bl/6 mice, the genetic ablation of LANCL2 did not modify fasting glycemia values but resulted in the reduction of glucose tolerance compared with wild-type siblings, as indicated by a significantly increased AUC of glycemia after an oral glucose load; however, LANCL2^−/−^ mice were still responsive to exogenous ABA, which significantly reduced the AUC of glycemia after glucose load, to values similar to those of wild-type animals (Figure 4) (Magnone M., unpublished results).

This result clearly indicates that the genetic ablation of LANCL2 negatively affects glucose tolerance. The fact that exogenous low-dose ABA (1 µg/Kg body weight) improved glucose tolerance in LANCL2^−/−^ mice suggests that another receptor can substitute for LANCL2 in the stimulation of muscle and AT glucose transport, although at higher ABA concentrations than those reached by the endogenous hormone (Magnone M., unpublished results). Indeed, intake of ABA at a dose of 1 µg/Kg body weight increases ABAp between 10 and 50 times compared to basal, endogenous levels in humans [15]. These high plasma concentrations, obtained by pharmacologic intervention, could activate a low-affinity receptor not normally participating in endogenous ABA signaling.

The identity of this receptor remains to be determined; however, the high sequence identity (54%) between LANCL2 and LANCL1 is highly suggestive of a role for LANCL1 as a second ABA receptor, in addition to LANCL2. These results suggest a redundancy in ABA-sensing molecules in mammals too, as occurs in plants, which could be expected given the strategic role of the hormone in the response to nutrients. Nonetheless, absence of LANCL2 negatively affects glucose tolerance in mice, indicating that the other ABA receptor(s) do not wholly substitute for this protein. Further studies are definitely needed to deepen our understanding of the physiology of mammalian ABA receptors, their tissue distribution and affinity for the hormone.

He et al. published a study on the triple knock out of LANCL1-2-3 in mice, demonstrating that these proteins are not involved in the synthesis of lanthionine, which conversely could have been hypothesized based on their homology with bacterial LanC enzymes. It would be informative to compare glucose tolerance and insulin sensitivity on the LANCL2 KO and on the double (LANCL1 + 2) KO [26].

## 2. Conclusions and Future Perspectives

In this review, we focused our attention on the metabolic function of the ABA/LANCL2 system on glycemic and lipidemic control, neglecting other important aspects of ABA physiology in mammals, such as its regulatory role in inflammation, for which we redirect the reader to the excellent review by Lievens et al. [44].

The results summarized in this review allow to draw some conclusions, but also provide the starting point for future investigations, both clinical and preclinical.

The significant beneficial effect of micrograms of oral ABA observed in volunteers with metabolic and morphometric parameters borderline with the metabolic syndrome allows to forecast similar positive results in trials of the ABA-containing nutraceutical on a higher number of prediabetic subjects and of subjects with the metabolic syndrome. The primary outcomes of this study would be the improvement of glucose and lipid tolerance and the reduction of body weight.

In addition, studies on murine model(s) of insulin-dependent diabetes could provide preclinical evidence supporting the use of oral low-dose ABA to improve glycemia control in combination with insulin. This result in turn would warrant clinical studies aimed at confirming whether ABA-containing nutraceuticals could represent an adjunctive therapy in insulin-dependent diabetic patients (both T1D and T2D), improving the daily glycemia profile, reducing blood lipids and contributing to the control of body weight by reducing the amount of insulin required for glycemic control.

The physiology and possible dysfunction of LANCL2 and of other ABA receptors still to be identified is another important area of research. Do LANCL2 mutations or constitutively low expression levels, particularly in skeletal muscle, predispose to “ABA resistance” and to diabetes? In this case, intake of the ABA-containing nutraceutical would provide the amount of ABA sufficient to increase ABAp 5- to 10-fold over endogenous levels, overtaking ABA resistance.

Another issue requiring further study is the identity of the ABA-producing cells in mammals. Results obtained on T1D patients seem to indicate that β-pancreatic cells are the main ABA producers, since ABAp is reduced by approximately 90% compared to values measured in healthy controls. In addition, rat insulinoma cells and human pancreatic islets release ABA after stimulation with GLP-1 [14]. In mice, a very high ABA concentration has been measured in the BAT, but not in the WAT, suggesting that BAT could be another source of endogenous ABA in mammals. The identification of the main ABA-releasing cells in humans is relevant to understand which physiological stimuli induce an ABA response in humans. In particular, if β-pancreatic cells were indeed the main ABA producers, the demise of these cells in T1D would compromise secretion of both hormones regulating glycemia, insulin and ABA, of which only one is currently replaced by therapy.

Finally, a field of exploration on mammalian ABA physiology which lies at the crossroad between metabolism and inflammation is the role of ABA in the central nervous system. The fact that brain has the highest ABA content among the various tissues, as first reported by Le Page-Degivry et al. [3], raises the possibility that ABA is produced and acts locally in the brain. ABA administration improves neuroinflammation and cognitive impairment and anxiety in rodents [45,46]. Interestingly, phaseic acid, the principal ABA metabolite in plants, is apparently endogenously produced in the brain and protects from ischemic injury by acting as a non-competitive inhibitor of glutamate receptors [47].
